# Predictive modeling of Persian walnut (*Juglans regia* L.) in vitro proliferation media using machine learning approaches: a comparative study of ANN, KNN and GEP models

**DOI:** 10.1186/s13007-022-00871-5

**Published:** 2022-04-11

**Authors:** Mohammad Sadat-Hosseini, Mohammad M. Arab, Mohammad Soltani, Maliheh Eftekhari, Amanollah Soleimani, Kourosh Vahdati

**Affiliations:** 1 Department of Horticulture, Faculty of Agriculture, University of Jiroft, Jiroft, Iran; 2 Department of Horticulture, College of Aburaihan, University of Tehran, Tehran, Iran; 3 Department of Irrigation and Draintage Engineering, College of Aburaihan, University of Tehran, Tehran, Iran; 4 Department of Horticultural Science, Faculty of Agriculture, Tarbiat Modares University (TMU), Tehran, Iran; 5 Department of Agronomy and Plant Breeding, Faculty of Agriculture, University of Jiroft, Jiroft, Iran

**Keywords:** Walnut in vitro propagation, Artificial neural network, Gene expression programming, *k*-nearest neighbors, Prediction model

## Abstract

**Background:**

Optimizing plant tissue culture media is a complicated process, which is easily influenced by genotype, mineral nutrients, plant growth regulators (PGRs), vitamins and other factors, leading to undesirable and inefficient medium composition. Facing incidence of different physiological disorders such as callusing, shoot tip necrosis (STN) and vitrification (Vit) in walnut proliferation, it is necessary to develop prediction models for identifying the impact of different factors involving in this process. In the present study, three machine learning (ML) approaches including multi-layer perceptron neural network (MLPNN), *k*-nearest neighbors (KNN) and gene expression programming (GEP) were implemented and compared to multiple linear regression (MLR) to develop models for prediction of in vitro proliferation of Persian walnut (*Juglans regia* L.). The accuracy of developed models was evaluated using coefficient of determination (R^2^), root mean square error (RMSE) and mean absolute error (MAE). With the aim of optimizing the selected prediction models, multi-objective evolutionary optimization algorithm using particle swarm optimization (PSO) technique was applied.

**Results:**

Our results indicated that all three ML techniques had higher accuracy of prediction than MLR, for example, calculated R^2^ of MLPNN, KNN and GEP vs. MLR was 0.695, 0.672 and 0.802 vs. 0.412 in Chandler and 0.358, 0.377 and 0.428 vs. 0.178 in Rayen, respectively. The GEP models were further selected to be optimized using PSO. The comparison of modeling procedures provides a new insight into in vitro culture medium composition prediction models. Based on the results, hybrid GEP-PSO technique displays good performance for modeling walnut tissue culture media, while MLPNN and KNN have also shown strong estimation capability.

**Conclusion:**

Here, besides MLPNN and GEP, KNN also is introduced, for the first time, as a simple technique with high accuracy to be used for developing prediction models in optimizing plant tissue culture media composition studies. Therefore, selection of the modeling technique to study depends on the researcher’s desire regarding the simplicity of the procedure, obtaining clear results as entire formula and/or less time to analyze.

## Background

The walnut is one of the most important nuts in the world. Persian walnuts (*Juglans regia* L.) are the only edible species of walnut which are widely grown for their nuts and timbers [1]. In general, walnut tree propagation is still mainly by using seeds rather than vegetative procedures which results in non-uniform nut quality and irregular yielding [2]. Therefore, in vitro propagation is used to overcome the mentioned problems. But walnuts are considered recalcitrant to in vitro culture which makes difficult the mass propagation of different genotypes while several micropropagation protocols have been published for different genotypes [3–11]. It has been proven that walnut micropropagation results are highly dependent upon genotype [7, 9–11]. In addition to genotype, the formulation of culture medium has a great impact on all micropropagation stages. Up to now, the [3] walnut (DKW) culture medium has been the most employed formulation for walnut tissue culture. Nevertheless, there are some researches reporting improved results using modified DKW or other formulations [6–8, 12–15].

However, to the best of our knowledge, no comprehensive study has been done on the balance of culture media components (mineral nutrients, plant growth regulators (PGRs) and vitamins) and their interaction together and with genotype on walnut in vitro performance to increase the efficiency of the micropropagation process by enhancing proliferation rate and reducing physiological disorders.

Predicting the interaction of mineral nutrients, PGRs, vitamins and genotype on the explant in vitro performance would involve modeling a very complex database, which is very problematic and time-consuming process using classic statistical analyses and needs accurate and advanced modeling procedures [16, 17]. Machine learning (ML) tools allow researchers to perceive the studied process and make proper decisions to develop optimal culture media [17]. In recent years, different ML models like neural networks [18–23] have been successfully applied for prediction and optimization of different plant tissue culture processes. In our previous studies, we described the ML hybrid techniques, combining artificial neural network (ANN) with genetic algorithm (ANN-GA) in *Pyrus* [24] and *Prunus* rootstocks [25–27], rootstocks gene expression programing (GEP) with GA (GEP-GA) [20] and particle swarm optimization (PSO) (GEP-PSO) [28] in *Pyrus* rootstocks as powerful data mining approaches, which allow modeling of complicated databases and finding the factors influencing a given response in micropropagation process.

ANNs are inspired by the functions of human brain [29]. The ANN [multi-layer perceptron neural network (MLPNN) and radial basis function neural network (RBFNN)] has revealed significant development in complex plant tissue culture systems [20, 24–27]. ANN does not require any previous knowledge regarding the creation or interrelationships between signals of input and output that is one of its profits [16]. Other benefits of ANN are prediction of the plant biomass [30], clustering the micropropagated plantlets and influencing growth and quality of the regenerated plants by controlling light, ventilation, CO_2_ and air temperature inside the culture containers which could be of ANN benefits [16].

GEP model is another ML-based optimization technique presented by [31] which comprises useful traits of both genetic programming (GP) and GA. This new model according to an evolving computer programs algorithm was used in our previous studies on *Pyrus* rootstocks micropropagation which precisely detected nonlinear and complicated relationships between input and output [20, 24].

Here, ANN and GEP are compared to *k*-nearest neighbors (KNN) method as one of the simplest machine learning techniques. The KNN technique recognizes the elements amongst the training samples that correspond “current” conditions maximum closely based on some predefined attributes: the neighbors. The prediction value is then specified from the groups of the next values of the neighbors [32]. Comparing to mathematical modeling, the KNN method involves no model development or confirmation and thus can be used without recombining data, contrasting in the case of common data-based models [33]. In spite of the potential advantages, no research has yet been done on the use of this technique in the area of plant micropropagation.

In our previous study [20], we compared the RBFNN and GEP in optimizing the in vitro culture media composition for pear rootstocks. Based on our results GEP was a significantly powerful and more precise technique than RBFNN in prediction of in vitro proliferation quantity and quality. So, GA technique was applied to optimize GEP models [20]. Nevertheless, GA optimized the level of inputs required for each specific output, distinctly. Consequently, in our recent study [28], in order to achieve a complete optimum formulation for culture medium, we compared two algorithms GEP and M5’ model tree, to predict the impacts of media minerals and PGRs on in vitro proliferation of pear rootstocks. We found that GEP showed a higher prediction precision than M5’ model tree. So, we optimized the GEP prediction models using multi-objective evolutionary optimization algorithms (MOEAs) including GA and PSO methods and compared to the mono-objective GA optimization procedure. The PSO optimized GEP prediction models made the best outputs in both rootstocks [28].

With MOEAs, inputs are evaluated as multi-objective optimization problems (MOPs) and the solutions specify the best probable balance between two reverse functions [34]. Recently, several mathematical methods have been used to solve MOPs, nonetheless the real MOPs applications are specifically nonlinear and also occasionally non-differentiable [35]. This has enhanced interest in metaheuristic methods, and among these procedures, MOEAs are of special interest. Here, PSO as an evolutionary computation technique was used for determining optimized culture media.

The aim of this study is to employ three soft computing methods namely MLPNN, GEP and KNN and to compare the accuracy of their prediction to multiple linear regression (MLR) technique as well as applying PSO algorithm with aim of predicting and optimizing walnut tissue culture media. Briefly, the new contributions of the present research are:

- Comparing the appropriateness of MLPNN, KNN and GEP nonlinear methods for modeling the impacts of mineral nutrients, PGRs and vitamins on *in vitro* culture of walnut.
- Constructing hybrid models in order to assess how Chandler and Rayen explants respond to the culture medium composition according to the new produced shoots attained from the Taguchi design.
- Finding the optimal composition of culture media to maximize the proliferation rate (PR) and minimize callus weight (CW), shoot tip necrosis (STN) and vitrification (Vit) by optimizing the developed model using PSO.

To our knowledge, this study is the first application of MLR, KNN, ANN, GEP and PSO methods for optimizing walnut tissue culture media. In addition, this work is the first use of KNN modeling procedure in plant tissue culture.

## Results

**Fig. 1:**
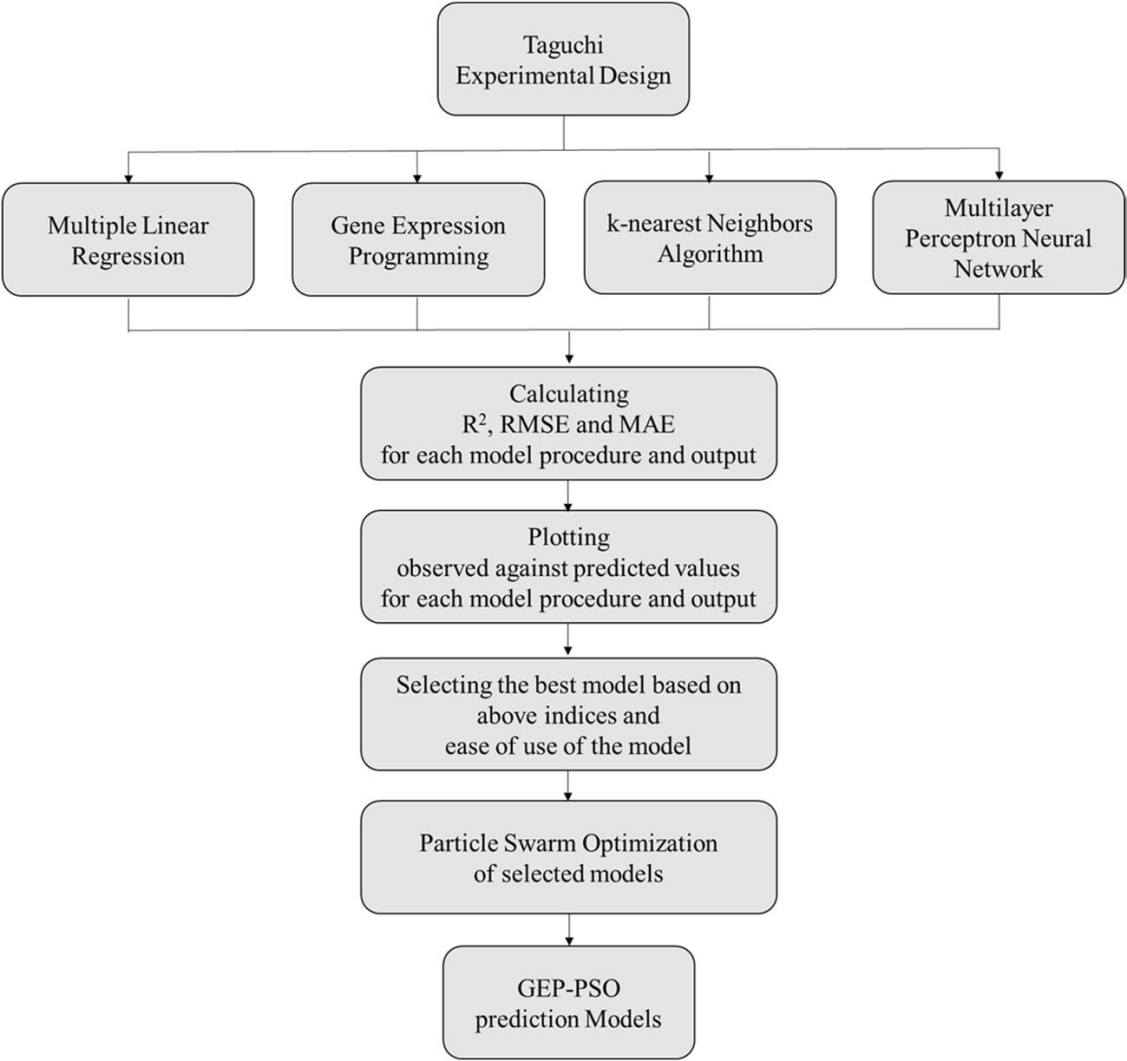
Schema of the techniques used to construct prediction models for Persian walnut in vitro culture media

Our models of the interaction of modifying inputs including nutrients, PGRs and vitamins on outputs including PR, CW, STN and Vit were developed using MLR, MLPNN, KNN and GEP techniques. Here, we assess the developed models’ performances through evaluating each modelling method precision to predict the composition of plant micropropagation media for walnut. After that, PSO optimization results of the selected modeling method is investigated to find the most efficient compositions of media for each considered trait. An outline of the techniques used here to achieve the most appropriate model is shown in Fig. 1.

### Comparison of modeling techniques performances

The mathematical equations attained from GEP method, which is showing the best estimate of the explant growth parameters, are shown in Table 1. Moreover, calculated statistics results for output variables (PR, CW, STN and Vit) related to the MLR, MLPNN, KNN and GEP models are given in Table 2. Unlike MLR, the trained MLPNN, KNN and GEP models of PR, CW, STN and Vit resulted in balanced statistic values for both the training and testing subsets (Table 2). For output variables (PR, CW, STN, and Vit) the calculated statistical values corresponding to the KNN, MLPNN and GEP models showed a considerably higher accuracy of prediction than for MLR models as calculated R^2^ for MLPNN, KNN and GEP vs. MLR models were: 0.672, 0.695 and 0.802 vs. 0.412 for PR of Chandler; 0.377, 0.354 and 0.428 vs. 0.178 for PR of Rayen; 0.923, 0.931 and 0.844 vs. 0.696 for CW of Chandler; 0.929, 0.930 and 0.839 vs. 0.276 for CW of Rayen; 0.855, 0.915 and 0.807 vs. 0.241 for STN of Chandler; 0.812, 0.831 and 0.808 vs. 0.341 for STN of Rayen; 0.974, 0.975 and 0.853 vs. 0.434 for Vit of Chandler; and 0.977, 0.978 and 0.891 vs. 0.299 for Vit of Rayen, respectively (Table 2).

**Table 1:** Constructed models using gene expression programming to predict explant growth traits in Persian walnut

Walnut	Equation
Chandler	$$\begin{aligned}PR=\left[X3-\left({\left(\mathrm{cos}\left(\frac{X8}{X1}\times 6.178\right)\right)}^{3}\times X5\times X6\right)\right]+\left[X6+\left(8.010-\left(\frac{\mathrm{cos}\left(X4\right)\times 0.989}{\mathrm{cos}\left(X8+X3\right)}\right)\right)\right]+\left[-3.539-\mathrm{sin}\left(\left(\left(5.549+X1\right)\times \left(-0.883\right)\times X4\right)+\left(X7-X9+\left(X2-3.579\right)\right)\right)\right]+\left[X4+\left(X7-\left(\mathrm{cos}\left(\left(\left(X2\times X1\right)\times \left(-8.741\right)\times X7\right)\times X1\times X5\right)\right)\right)\right]\end{aligned}$$
	$$\begin{aligned}CW=\left[4.719\times \frac{X1}{{\left(5.715\right)}^{X1}-\left({X8}^{4.879}+\frac{6.250}{X8}\right)}\right]+\left[\mathrm{cos}\left(-4.432-\left(\left(X9-X3-0.208\right)\times \left({X5}^{X5}\times 10.590\right)\right)\right)\right]+\left[\left(\frac{7.082-6.718X4}{{\left(X6-6.287\right)}^{3}}\right)\times \left(X3-X7X4\right)\right]+\left[{\mathrm{cos}}^{2}\left(X9-{\left(\mathrm{cos}\left(\left(4.957-X2\right)-\mathrm{cos}\left(X4\right)\right)\right)}^{4}\right)\right]\end{aligned}$$
	$$\begin{aligned}STN=\left[-5.448\times \mathrm{sin}\left(X7\times \left(X7+\left(\left(X5+5.490\right)\times \left(X1X4\right)\right)\right)\right)\right]+\left[7.232\times \left(3.087-\left(\left({\mathrm{sin}}^{2}\left(X2-X6\right)\right)\times \mathrm{sin}\left(X5-3.272\right)\right)\right)\right]+\left[\left(X7\times \left(X7-X3+X2\right)\right)\times \left(X8+\mathrm{sin}\left(3.793X9\right)\right)\right]+\left[X6\times \left(X9+\left(X8+\left(X7\times \left({X8}^{2}-6.451\right)\right)\right)\right)\right]\end{aligned}$$
	$$\begin{aligned}Vit=\left[\mathrm{sin}\left(X6-\left(X2\times \left({1.392X1}^{{F8}^{3}}+1.334X9\right)\right)\right)\right]+\left[\mathrm{sin}\left(X1\right)+X1+{X1}^{3}+\left(\frac{X9}{X7}\times 2X8\right)\right]+\left[\mathrm{cos}\left(\left(\left(2X1+X2\right)\times \left(X1+X4-{X3}^{3}\right)\right)\right)\right]+\left[{X5}^{{F9}^{\left(\left(\frac{6.882-X5}{X2+X5}\right)\times \left(\mathrm{sin}\left({X7}^{X1}\right)\right)\right)}}\right]\end{aligned}$$
Rayen	$$\begin{aligned}PR=\left[\mathrm{sin}{\left(\mathrm{cos}\left({X2}^{3}\times \left(\left(X4+X6\right)\times \frac{-2.567}{X9}\right)\right)\right)}^{9}\right]+\left[X1+\left(\mathrm{sin}\left(\left(\mathrm{cos}\left(X3\times X1\right)\right)-\left(\mathrm{sin}\left({X3}^{X9}\right)\right)\right)+3.955\right)\right]+\left[\left(X6\times \frac{\mathrm{sin}\left(X5\right)}{\mathrm{sin}\left(X7\right)+{X5}^{X9}}\right)+X3\right]+\left[\mathrm{sin}\left(X5\times \left({X8}^{39.898}\right)\right)-0.108X7\right]\end{aligned}$$
	$$\begin{aligned}CW=\left[\mathrm{sin}\left(\frac{\mathrm{sin}\left(\left(\frac{X9}{X6}+1.351X7\right)-\left(\frac{\mathrm{cos}\left(X5\right)}{-2.359}\right)\right)}{-1.721}\right)\right]+\left[\mathrm{sin}\left(\mathrm{cos}\left({\left({\left(\mathrm{sin}\left(X7\right)+0.566\right)}^{2}\right)}^{\left({X6}^{3}\times \frac{X3}{-7.132}\right)}\right)\right)\right]+{\left[\mathrm{cos}\left(0.089+\left(\mathrm{cos}\left(\frac{X6}{X4-3.919}\right)\right)\right)\right]}^{X2}+\left[\mathrm{sin}\left(\left(X1-X5+0.282\right)\times \left(\frac{\mathrm{cos}\left(X1\right)}{-1.095-X8}\right)\right)\right]\end{aligned}$$
	$$\begin{aligned}STN=\left[6.002\times \left(\left(\mathrm{cos}\left(\left(3.862X3+X9-X1\right)\times \left(\mathrm{sin}\left(X7\right)\right)\right)\right)-X4\right)\right]+{\left[X2\times \left(X3+\mathrm{cos}\left(X2\times \left(X8-X8+3.742\right)\right)\right)\right]}^{2}+\left[\left({\left(X9-4.711\right)}^{2}-\left(X6\times X4\times X1\right)\right)+45.170\right]+\left[\frac{\mathrm{cos}\left(F1\right)}{\left(X5-1.460\right)-\left(\mathrm{cos}\left(X2\right)\times {X2}^{X1}\right)}\right]\end{aligned}$$
	$$\begin{aligned}Vit=\left[\frac{X4}{\mathrm{sin}\left(F1\times \left(9.069+F4F6-F4F3\right)\right)}\right]+\left[X2+\left({X1}^{3}-\left({\left(X8+X5\right)}^{-2.497X9}+X1\right)\right)\right]+\left[8.504+\left({X7}^{X7}\times \left(\left({X9}^{-1.651}-X6\right)\times \left(X3-X6\right)\right)\right)\right]+\left[\frac{X8\times \left(\mathrm{sin}\left(X2-X6\right)\times \left(X9\times \left(X2-X5\right)\right)\right)}{\mathrm{sin}\left(X7\right)}\right]\end{aligned}$$

X1–X9: are input factors as presented in Table 3

**Table 2:** Evaluation of different developed models using various statistics for PR, CW, STN, and Vit of Persian walnut through *in vitro* proliferation

Walnut	Output	Model	Train Set ^*^			Test Set		
			RMSE	MAE	R^2^	RMSE	MAE	R^2^
Chandler	PR	MLR	2.840	1.986	0.264	3.313	2.270	0.412
		KNN (4) ^**^	1.121	0.814	0.909	1.756	1.257	0.672
		MLPNN (9-40-1) ^***^	1.093	0.796	0.913	1.658	1.186	0.695
		GEP	1.496	1.093	0.805	1.711	1.299	0.802
	CW	MLR	0.225	0.163	0.640	0.190	0.146	0.696
		KNN (4)	0.068	0.046	0.955	0.094	0.063	0.923
		MLPNN (9–15-1)	0.063	0.045	0.960	0.083	0.058	0.931
		GEP	0.113	0.086	0.886	0.124	0.105	0.844
	STN	MLR	7.628	5.787	0.315	7.827	6.614	0.241
		KNN (5)	3.324	2.432	0.867	3.529	2.767	0.855
		MLPNN (9–30-1)	2.777	1.994	0.917	2.660	2.026	0.915
		GEP	3.980	3.080	0.814	3.910	3.156	0.807
	Vit	MLR	6.189	4.930	0.435	6.909	5.538	0.434
		KNN (3)	0.992	0.735	0.986	1.232	0.960	0.974
		MLPNN (9–30-1)	0.993	0.699	0.988	1.184	0.938	0.975
		GEP	2.461	1.901	0.906	3.371	2.584	0.853
Rayen	PR	MLR	1.310	1.041	0.181	1.388	1.191	0.178
		KNN (4)	1.049	0.843	0.471	1.159	0.970	0.377
		MLPNN (9–12-1)	1.008	0.832	0.512	1.169	1.000	0.358
		GEP	1.039	0.858	0.459	1.178	0.992	0.428
	CW	MLR	0.400	0.319	0.286	0.460	0.382	0.276
		KNN (4)	0.069	0.050	0.949	0.078	0.057	0.929
		MLPNN (9–30-1)	0.067	0.049	0.952	0.078	0.056	0.930
		GEP	0.207	0.160	0.853	0.223	0.178	0.839
	STN	MLR	12.757	10.150	0.046	10.170	8.273	0.341
		KNN (5)	2.671	2.087	0.821	3.378	2.805	0.812
		MLPNN (9–30-1)	2.574	2.47	0.837	3.224	2.606	0.831
		GEP	4.916	3.822	0.858	5.238	4.242	0.808
	Vit	MLR	7.033	5.383	0.370	7.101	5.627	0.299
		KNN (4)	1.228	0.948	0.980	1.211	1.022	0.977
		MLPNN (9–20-1)	1.197	0.909	0.982	1.192	0.975	0.978
		GEP	2.849	2.235	0.899	2.937	2.452	0.891

^*^ Average of tenfold cross validation

^**^ The number of neighbors (k) leading to the best performance

^***^ The MLPNN architecture (inputs—hidden layers—outputs)

**Fig. 2:**
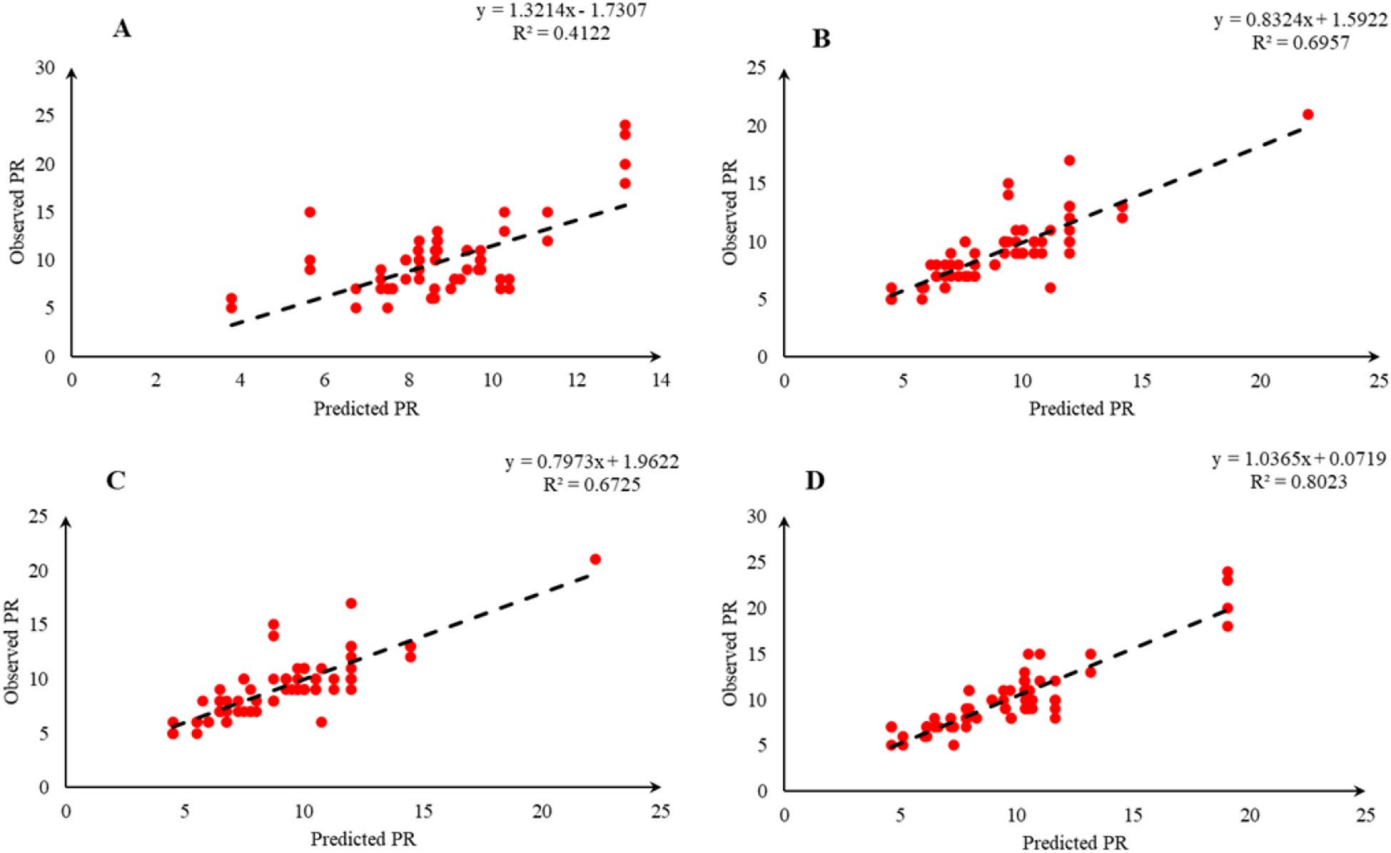
Observed vs. predicted values of proliferation rate (PR) related to **A** multiple linear regression (MLR); **B** multi-layer perceptron neural network (MLPNN); **C** *k*-nearest neighbors (KNN); **D** gene expression programming (GEP) developed models (n = 224) for walnut cv. Chandler

**Fig. 3:**
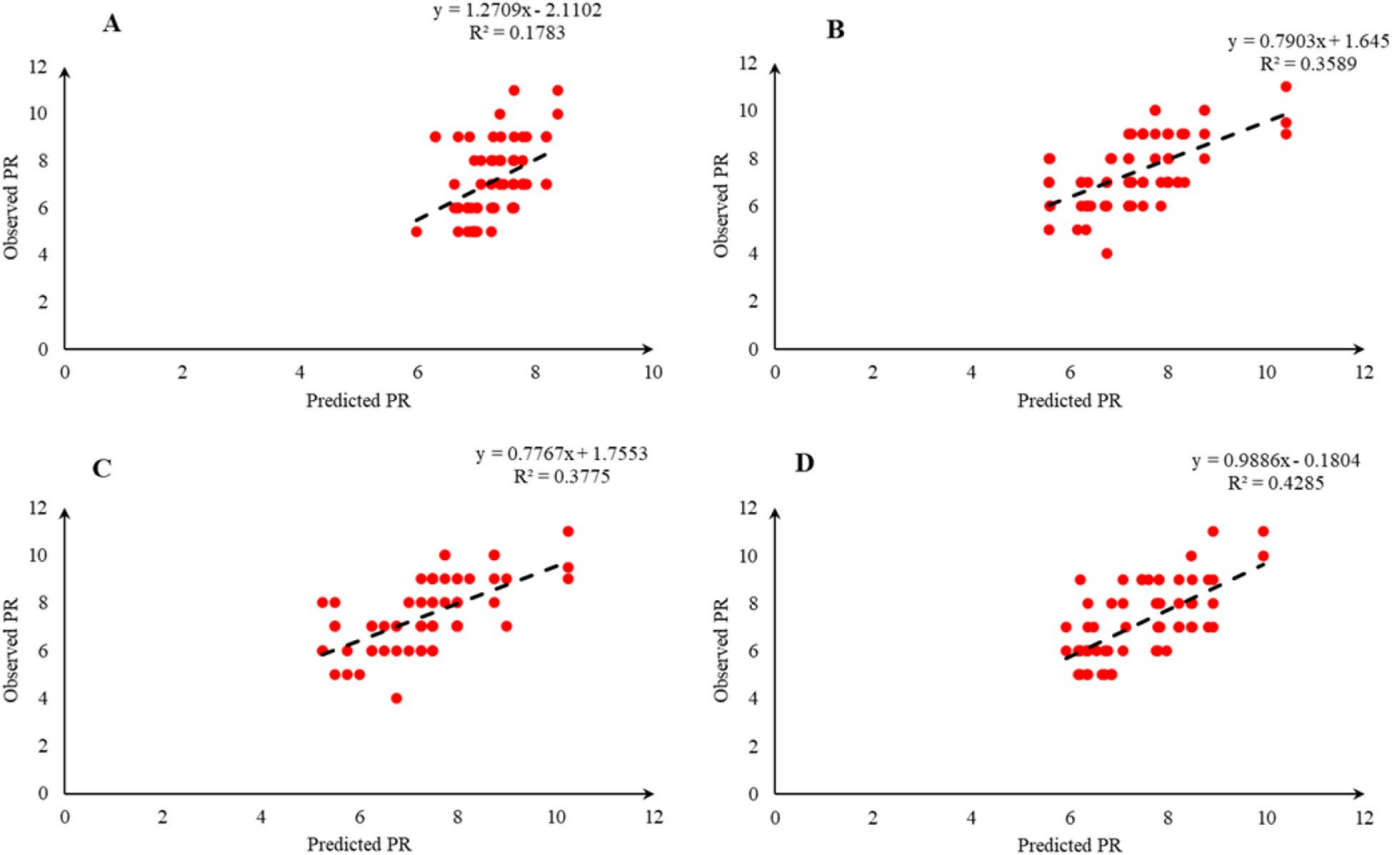
Observed vs. predicted values of proliferation rate (PR) related to **A** multiple linear regression (MLR); **B** multi-layer perceptron neural network (MLPNN); **C** *k*-nearest neighbors (KNN); **D** gene expression programming (GEP) developed models (n = 224) for walnut cv. Rayen

**Fig. 4:**
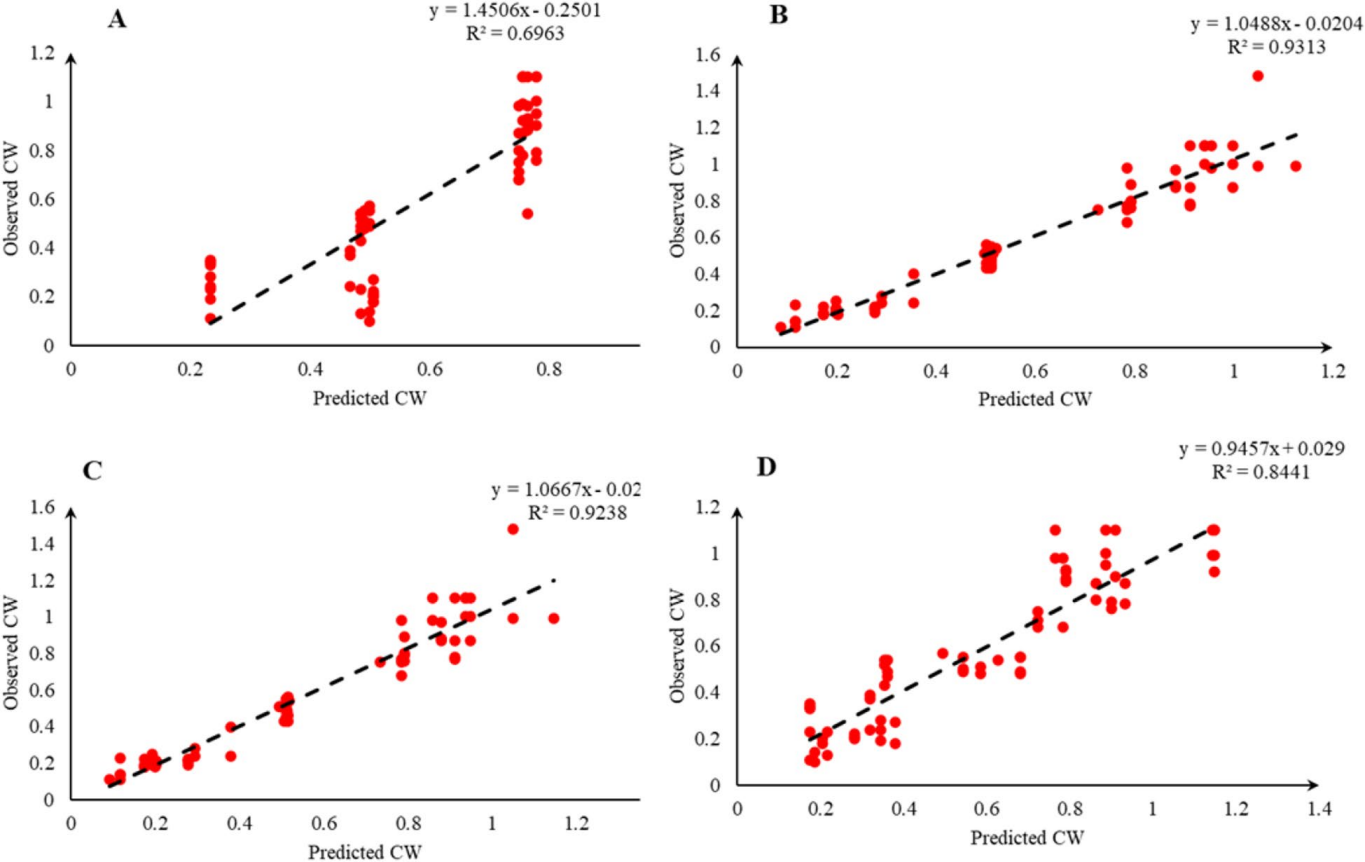
Observed vs. predicted values of callus weight (CW) related to **A** multiple linear regression (MLR); **B** multi-layer perceptron neural network (MLPNN); **C** *k*-nearest neighbors (KNN); **D** gene expression programming (GEP) developed models (n = 224) for walnut cv. Chandler

**Fig. 5:**
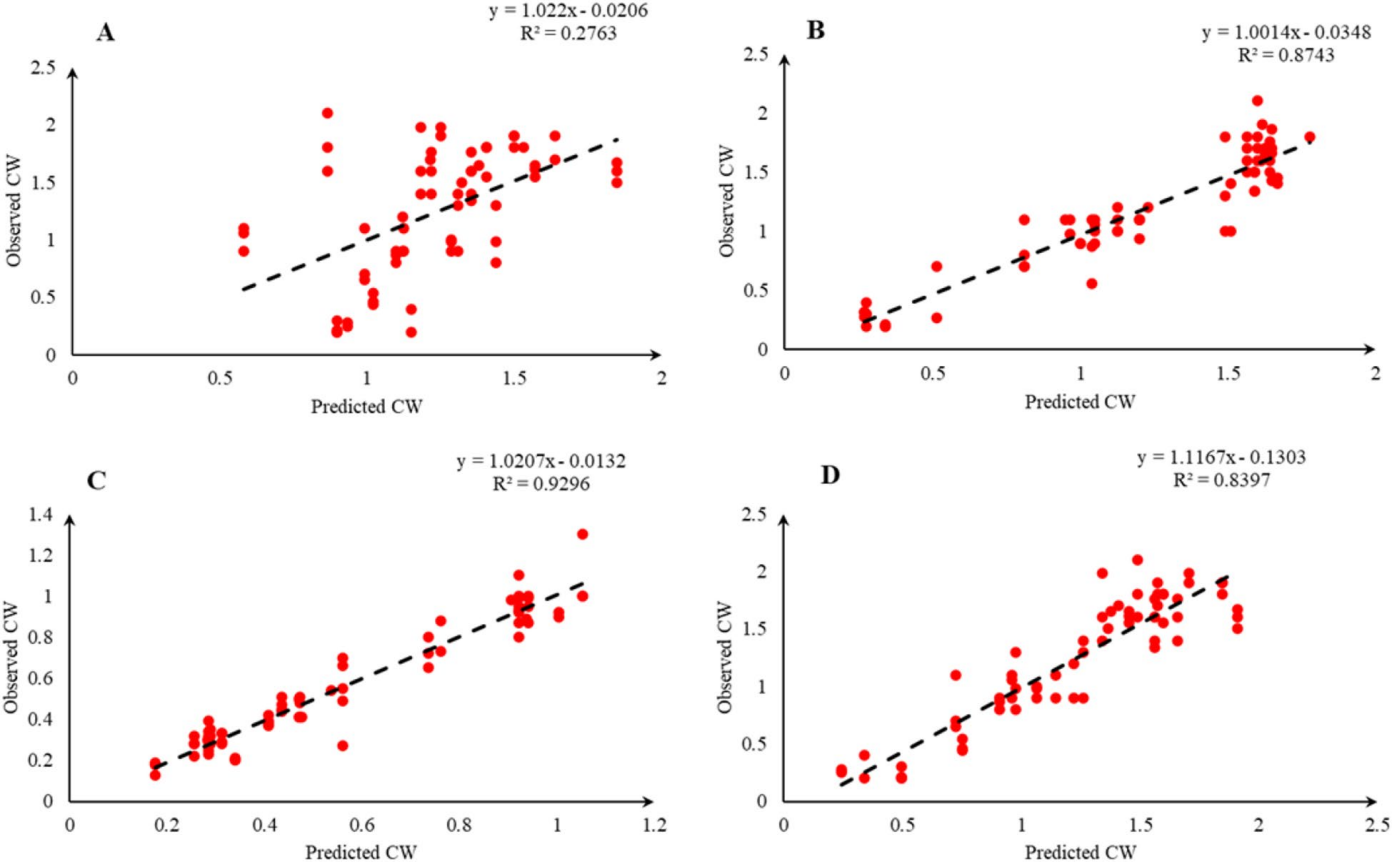
Observed vs. predicted values of callus weight (CW) related to **A** multiple linear regression (MLR); **B** multi-layer perceptron neural network (MLPNN); **C** *k*-nearest neighbors (KNN); **D** gene expression programming (GEP) developed models (n = 224) for walnut cv. Chandler

**Fig. 6:**
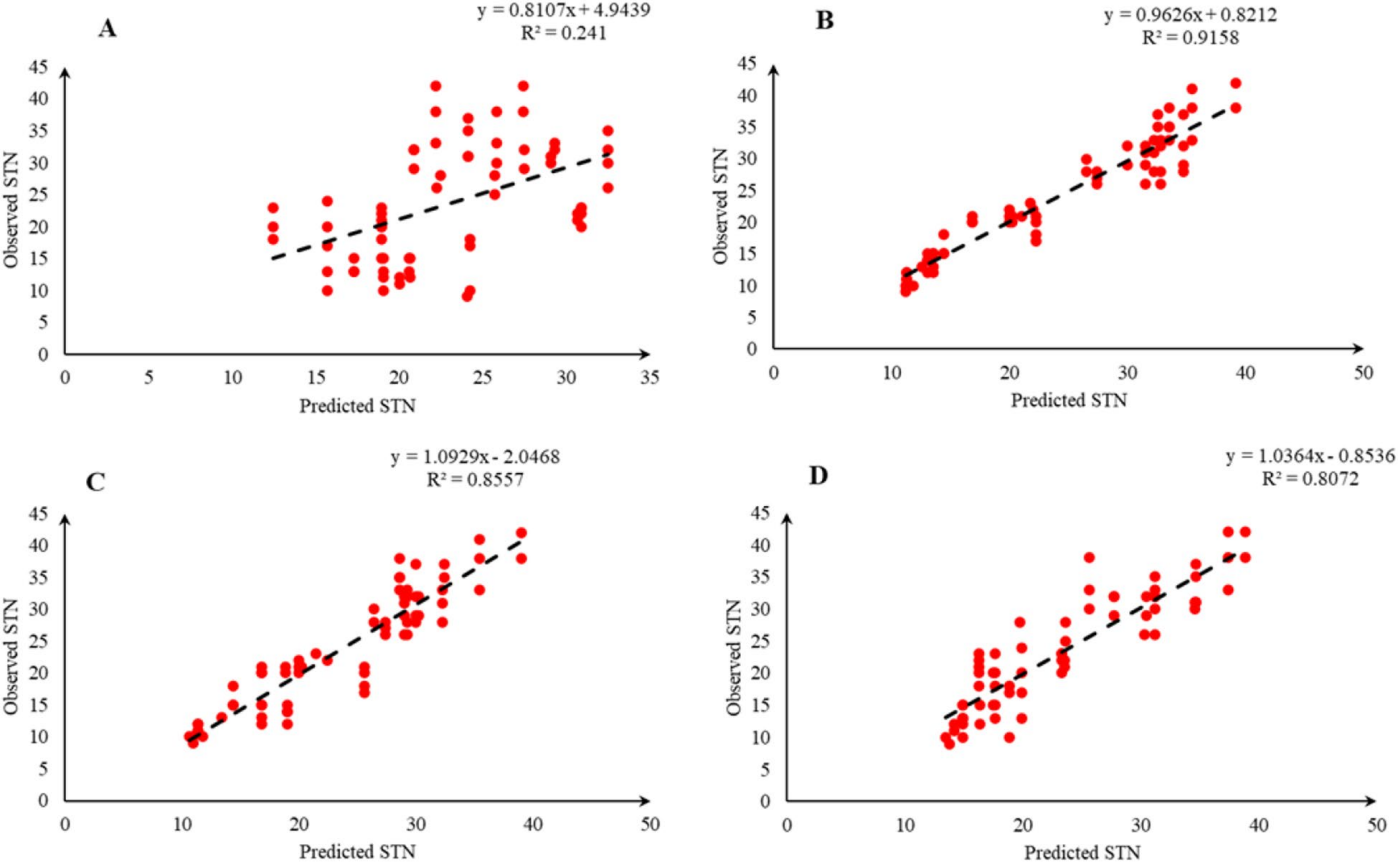
Observed vs. predicted values of shoot tip necrosis (STN) related to **A** multiple linear regression (MLR); **B** multi-layer perceptron neural network (MLPNN); **C** *k*-nearest neighbors (KNN); **D** gene expression programming (GEP) developed models (n = 224) for walnut cv. Chandler

**Fig. 7:**
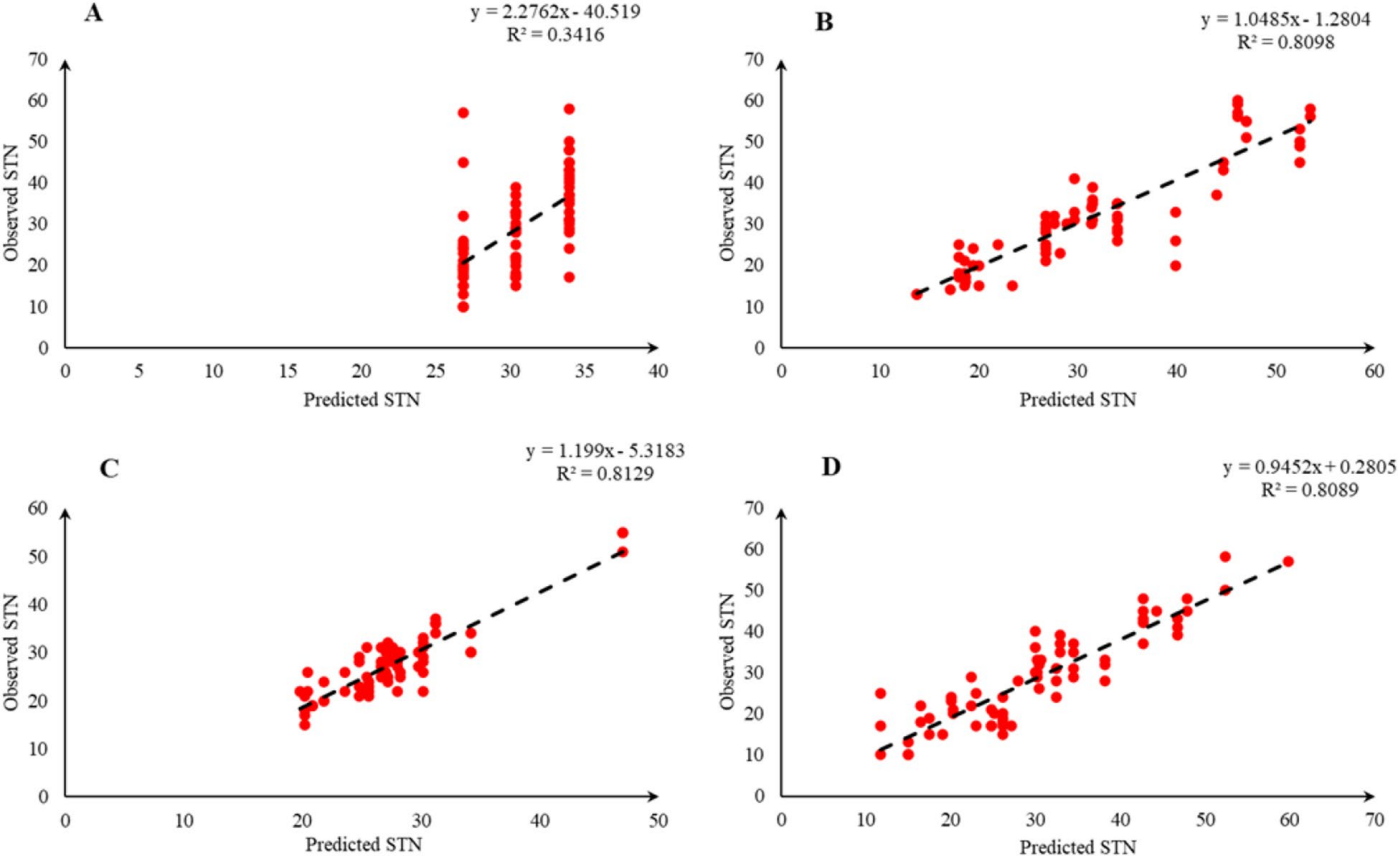
Observed vs. predicted values of shoot tip necrosis (STN) related to **A** multiple linear regression (MLR); **B** multi-layer perceptron neural network (MLPNN); **C** *k*-nearest neighbors (KNN); **D** gene expression programming (GEP) developed models (n = 224) for walnut cv. Rayen

**Fig. 8:**
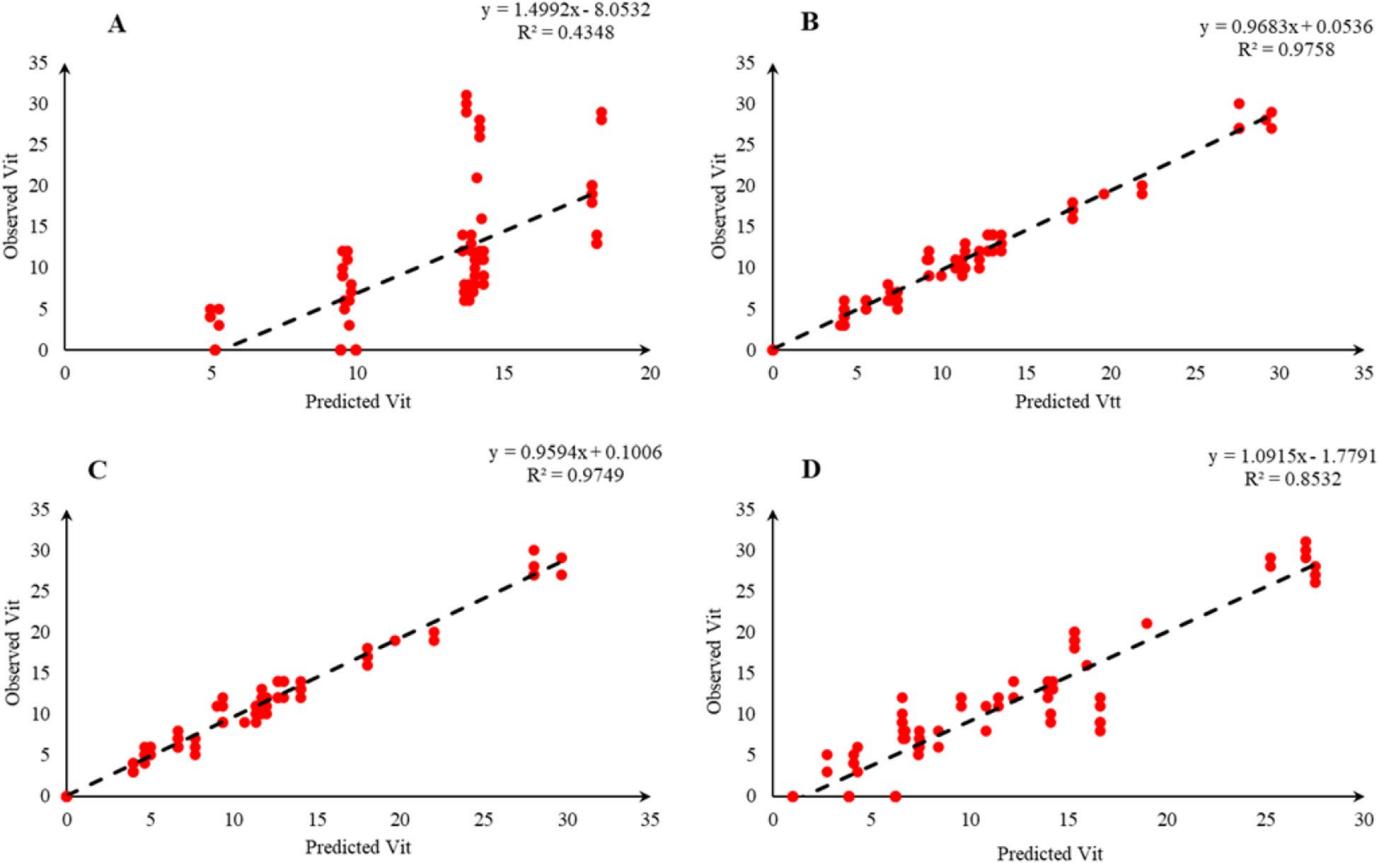
Observed vs. predicted values of vitrification (Vit) related to **A** multiple linear regression (MLR); **B** multi-layer perceptron neural network (MLPNN); **C** *k*-nearest neighbors (KNN); **D** gene expression programming (GEP) developed models (n = 224) for walnut cv. Chandler

**Fig. 9:**
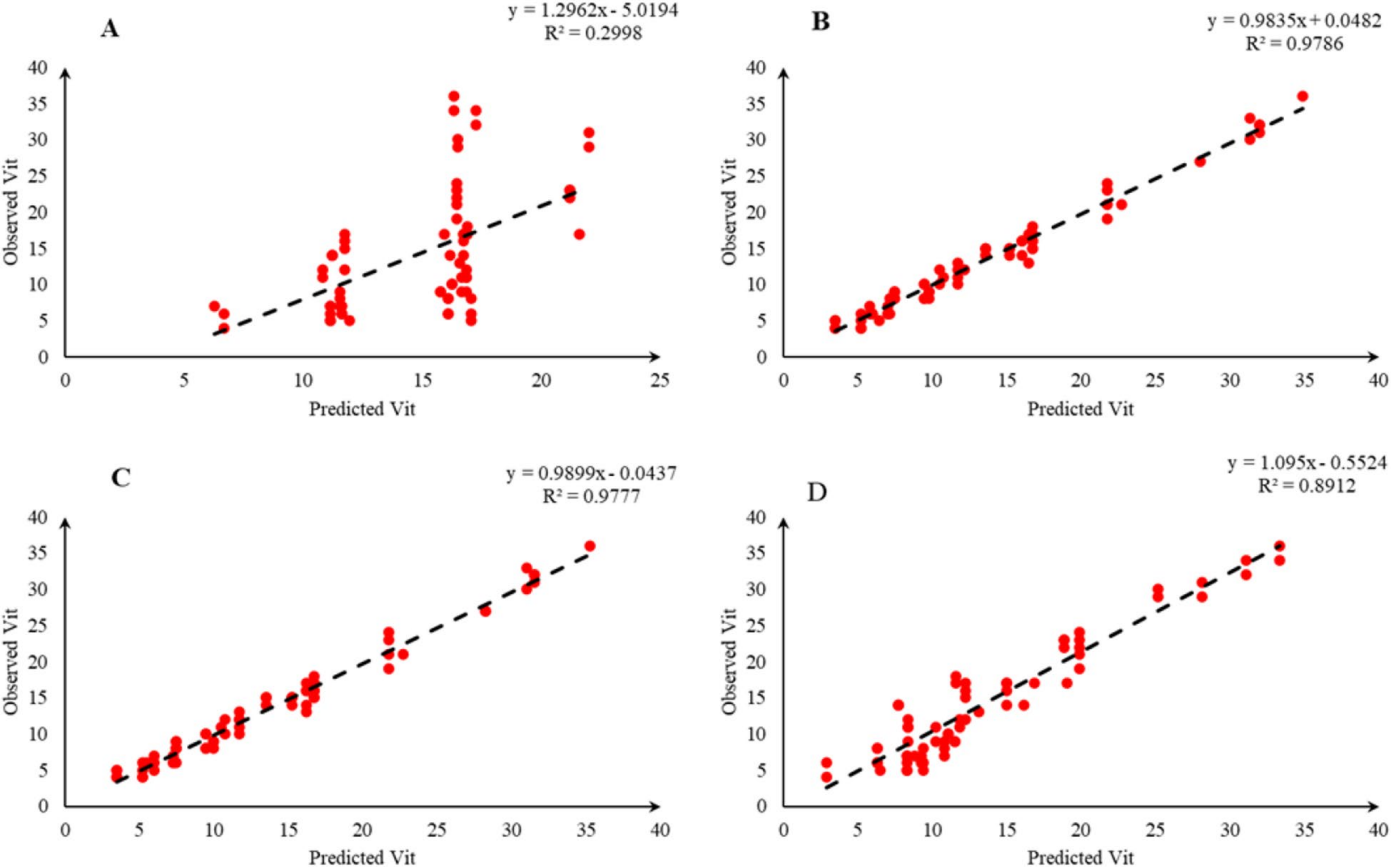
Observed vs. predicted values of vitrification (Vit) related to **A** multiple linear regression (MLR); **B** multi-layer perceptron neural network (MLPNN); **C** *k*-nearest neighbors (KNN); **D** gene expression programming (GEP) developed models (n = 224) for walnut cv. Rayen

Comparison of the observed and predicted values of outputs may explain the performance of the developed models according to the studied inputs. A high squared correlation coefficient fitting technique was used to produce plots according to the constructed models derived, to show how each of the four outputs varied as the concentration of media components changed. The plots may be helpful to understand the complete relationship between media components and responses, and to assess the multiple effects of modifying the media components in the DKW medium. The predicted MLR, MLPNN, KNN and GEP models diagrams vs. observed values for the PR, CW, STN and Vit are shown in Figs. 2, 3, 4, 5, 6, 7, 8 and 9. Comparing the fitted simple regression lines of the MLR with ML models showed that MLR resulted in the lowest accordance between the observed and predicted values regarding all considered outputs so that calculated R^2^ for MLPNN, KNN and GEP vs. MLR were: 0.696, 0.672 and 0.802 vs. 0.412 for PR of Chandler (Fig. 2); 0.178, 0.359, 0.377 and 0.428 for PR of Rayen (Fig. 3); 0.696, 0.931, 0.924 and 0.844 for CW of Chandler (Fig. 4); 0.276, 0.874, 0.930 and 0.840 for CW of Rayen (Fig. 5); 0.241, 0.916, 0.856 and 0.807 for STN of Chandler (Fig. 6); 0.342, 0.810, 0.813 and 0.809 for STN of Rayen (Fig. 7), 0.435, 0.976, 0.975 and 0.853 for Vit of Chandler (Fig. 8); and 0.300, 0.979, 0.978 and 0.891 for Vit of Rayen (Fig. 9), respectively. Therefore, the ML models were able to accurately predict the outputs while the MLR developed models were not able to describe extensive diversity of growth parameters owing to the studied variables interaction, that may hide the effects of media components. Figures 2, 3, 4, 5, 6, 7, 8 and 9 may be helpful for realizing the complete relationship between media components and responses, and assessing the combined impacts of modifying the DKW medium components.

According to the results presented in Table 2 and Figs. 2, 3, 4, 5, 6, 7, 8 and 9 as well as the above-mentioned results, MLPNN, KNN and GEP models performed accurately in predicting the effect of media components on in vitro performance of Persian walnut. So, in order to select one of these ML modeling techniques to be optimized and achieve final models for in vitro proliferation of Persian walnut, we considered the ease of using model by the end user. In other words, although MLPNN and KNN performed relatively well, none of these models offer explicit mathematical expression. Unlike MLPNN and KNN methods which produce black-box models, GEP can provide the researchers with an opportunity to optimize the extractive equations (optimal values of the variables) by generating explicit mathematical equations between the independent variable and the dependent variable and can be used as an equation for the pre-test stages (initial phase of the study) in designing and developing of their studies. Hence, we selected GEP models to be optimized and achieve proliferation media formulations of Chandler and Rayen.

### Optimization of GEP models

Consequently, to achieve the optimized medium resulting in the highest PR and the lowest CW, STN and Vit in walnut, we optimized developed GEP models by using multi-objective PSO technique.

The optimized amounts of the studied factors and the predicted values of growth parameters by the GEP models are shown in Table 3. The PSO optimization of the GEP models revealed that media containing 1.76 × NH_4_NO_3_, CaNO_3_ and ZnNO_3_, 1.67 × KNO_3_, 0.96 × K_2_SO_4_, 0.66 × MgSo_4_, MnSo_4_ and CuSo_4_, 2.35 × KH_2_PO_4_, H_3_BO_3_ and Na_2_MoO_4_, 1.64 × FeEDDHA and 1.89 × Thiamine, Nicotinic acid and Glycine concentrations in DKW medium, 0.67 mg/l BAP and 1.30 mg/l TDZ and 1.30 mg/l IBA could lead to optimal PR (23.54), CW (0.12), STN (2.23) and Vit (9.95) in Chandler and media containing 0.73 × NH_4_NO_3_, CaNO_3_ and ZnNO_3_, 0.69 × KNO_3_, 0.94 × K_2_SO_4_, 0.64 × MgSo_4_, MnSo_4_ and CuSo_4_, 0.83 × KH_2_PO_4_, H_3_BO_3_ and Na_2_MoO_4_, 1.35 × FeEDDHA and 1.52 × Thiamine, Nicotinic acid and Glycine concentrations in DKW medium, 0.67 mg/l BAP and 1.23 mg/l TDZ and 1.23 mg/l IBA could result in optimal PR (24.57), CW (0.64), STN (12.48) and Vit (3.04) in Rayen.

**Table 3:** Multi-objective PSO optimization of GEP models to achieve the highest quantity and quality through in vitro proliferation of walnut

Walnut	Medium	inputs									Outputs			
		NH4NO_3_, CaNO_3_, ZnNO_3_	KNO3	K2SO4	MgSo4, MnSo4, CuSo4	KH2PO4, H3BO3, Na2MoO4	FeEDDHA	Thiamine, Nicotinic acid, Glycine	BAP (mg/l)	TDZ, IBA (mg/l)	PR	CW (g)	STN	Vit
Chandler	Optimized	1.76	1.67	0.96	0.66	2.35	1.64	1.89	0.67	1.30	23.54	0.12	2.31	9.95
	DKW	1	1	1	1	1	1	1	1	0.1	7.12	0.3	10.75	0
Rayen	Optimized	1.78	0.85	0.93	1.43	1.95	1.59	0.53	0.99	0.52	7.42	0.70	11.29	12.99
	DKW	1	1	1	1	1	1	1	1	0.1	6.25	0.5	23.75	16.12

## Discussion

Walnuts as one of the important woody plants are considered recalcitrant to in vitro culture in which genetic determinism besides other factors such as media components makes more complicated different stages of micropropagation, as well. In the present study, three different ML modeling approaches along with PSO optimization algorithm were applied to determine and predict the effect of genotype and the media formulation throughout the proliferation of walnut. Walnut micropropagation can be improved by involving different physiological disorders in modeling and optimization processes. The incidence of physiological disorders through micropropagation of walnut has not been comprehensively investigated. Different studies on walnut tissue culture have been focused on introducing some chemicals like phloroglucinol and FeEDDHA to DKW or [36] (MS) basal media [11, 15], supplementing media with various concentrations of different PGRs [37–39], removing agar [39], ventilation and reducing sucrose concentration [40], but a few of the studies focused on media components, including mineral nutrients [9, 41], vitamins and PGRs [39] interaction on proliferation quality and quantity.

Here, we concentrated on increasing PR and reducing important abnormalities occurring during this phase, by recording data associated to several designed experiments. The subsequent database including a range of concentrations of each component in culture media allows simultaneous evaluation of the impacts of all minerals, vitamins and PGRs used in media as well as genotype on the explant growth indices only through the ML tools.

Machine learning as a powerful tool has been effectively applied in plant biology studies [42, 43] including plant tissue culture data analysis and accurate prediction of optimal in vitro culture media composition [20, 24–28]. The development of *in vitro* plant tissues is controlled by minerals, vitamins and PGRs in the culture media. To achieve maximum explant performance, the prediction of the most efficient media composition is highly useful since the optimization of the type and concentration of minerals, vitamins and PGRs in media is a time-consuming, expensive and laborious job [9, 41].

In our previous studies, we successfully performed constructing neural models using ANN technique to study the effects of different combinations of minerals and PGRs on in vitro proliferation and rooting of G × N15 *Prunus* rootstock [25–27]. Our study on comparing ANN with MLR modeling to forecast the optimum concentrations of macronutrients for OHF 69 and Pyrodwarf *Pyrus* rootstocks in vitro media showed ANN as a precise and promising technique [24]. The important benefit of ANN-based methods is that they do not need a prior identification of proper fitting function consequently; they have an overall approximation ability to calculate all kinds of non-linear functions in practice. This trait may help the modeler to develop the most possible precise model. Despite the fact that ANN is a good alternative for MLR, it does not provide us any equations including the relationships between input and output variables. Moreover, the ANN technique needs a time-consuming process of trial and error to find network parameters like number of neurons and hidden layers [44–46]. ANNs as the most extensively used ML model, can efficiently solve different multivariate, non-linear and nonparametric problems via an unidentified ‘‘black box” training [47]. Nevertheless, there are also some drawbacks with ANN “black box” nature [48]. In general, ANN is unable to clarify its logical process and this constraint makes ANN application unfriendly in natural science studies, as it can just simulate the change process according to experimental data, without helping us to understand the reason of the change.

Considering these restrictions in using ANN models, in another study on *Pyrus* rootstocks in vitro proliferation [20] we compared the power of GEP technique to ANN (RBFNN) and MLR in predicting the optimal media. RBFNN and GEP exhibited higher performance precision towards the MLR, and the GEP resulted in the most precise model as well as being practical [20]. In our recent research [28], we used two algorithms, GEP and M5’ model tree to overcome the ANN method weaknesses and simplify forecast of the media components interactions on in vitro proliferation of *Pyrus* rootstocks. Again, we found GEP as a more accurate technique than M5’ model tree [28].

Consequently, in the present study, we applied GEP as the most precise modeling procedure found by [20, 28], MLPNN as an ANN technique that its models are easier to give precise prediction than RBFNN when input data are randomly distributed [49] and KNN as one of the simplest machine learning approaches which can also be used for regression problems [50]. The MLR was also applied as a linear modeling method to be compared with above-mentioned ML procedures in predicting the optimum in vitro proliferation media composition of walnut to achieve the most appropriate outcomes. The accuracy of the developed prediction models was evaluated using MAE, RMSE and R^2^ statistics and correlation coefficient between observed and predicted values of each output. To our knowledge, KNN algorithms have not ever been applied to predict the plant tissue culture media composition. The advantage of KNN algorithm is that it does not require specific assumptions about the predictors’ distribution. The samples of KNN are classified according to the *k* neighbor responses mean values in a space of predictor [51]. The examples of training are defined by n traits. Each example means a point in a space with n-dimension. So, all examples of training will be kept in a space with the pattern on n-dimension. Here, the number of neighbors (*k*) leading to the best results for each model are presented in Table 3.

A key advantage of GP-based procedures such as GEP, toward other methods is that they do not need any hypothesis for preceding form of the relationship to produce prediction equations. GP and its deviations have been applied in many researches to find any complicated relationships which fit different experimental data [52–54]. An individuals’ population is employed in this technique and afterwards, better individuals are chosen by using genetic variations and fitness function. The genetic variations are introduced by genetic operators. Machine learning approaches including GEP have been programed to learn the variables̛ relationships in data collections. GEP difference with GA and GP as its precursors is in the method of individual programming so that in GEP, individuals are programmed as chromosomes i.e. fixed length linear strings which are presented finally as a simple diagram called expression tree. Whereas, in GA and GP, individuals are expressed, as nonlinear entities with different shapes (parse trees) and sizes and chromosomes, respectively. One of the GEP strengths over GA and GP is that genetic operators work very simple at the level of chromosome in GEP making development of genetic diversity. GEP unique, multi-genic nature is another important point which allows more complicated programs with multiple sub-programs to be developed. The advantages of both GA and GP are collected in GEP, whereas some of their constraints are met [55].

Based on our results presented in Table 2, KNN, MLPNN and GEP models were much more accurate than MLR. On the other hand, in most cases, the MLPNN method provided better fit calculation than KNN and GEP. But based on the results of our aforementioned studies [20, 28], the optimized GEP method provides better fit calculation than other approaches. Furthermore, GEP is preferred over ANN models, as ANN is a black-box model, whereas GEP explains the constructed prediction models with mathematical Eqs. [54].

Through the previous years, GEP has been applied extensively in other areas because of its high efficiency and effectiveness. GEP applications are so wide and are rapidly enhancing [55]. GEP is one of the most effective function mining algorithms which has been widely used in classification, pattern recognition, prediction, and other research areas. This algorithm can mine an ideal function to deal with further complex tasks [56]. GEP has been used to determine the quality and stress of water on lakes or rivers as a result of the wastewater pollutants [31]. The problem of missing values in data set due to the measurement conditions can simply be solved by employing GEP [31]. Results based on actual data set confirmed that the multiple GEP and fuzzy expert system outperforms detection methods in medical field by attaining high prediction precision [57].

Our previous studies [20, 24, 28] on pear rootstocks using ML-based modeling showed that there are different responses to the concentrations of macronutrients and PGRs based on genotypes, as we found here in Persian walnut varieties. Regarding the complex interactions, detection of the optimum levels of minerals and PGRs for a certain plant genotype is complicated [58]. Furthermore, the incidence of physiological disorders like Vit and STN throughout the proliferation phase of walnut needs improvement of media for optimal growth of explants. Constructing optimized and effective media by using authentic mathematical modeling and optimization methods have been performed previously on different plant species [17, 20, 24–28, 59–61]. Here, we consequently suggested use of ML-based modeling to recognize concentrations of minerals and PGRs that would maximize PR while minimizing CW, STN and Vit [24]. As we found here (Table 3), our previous results on pear [20, 24] showed that ANN prediction models had higher precision than MLR models and MLR could not be a trustworthy method for assessing nonlinear or non-polynomial relationships among variables.

It has been revealed from our recent study on pear rootstocks micropropagation [28] that the most efficient optimization method for optimizing GEP models was multi-objective PSO. Therefore, here, we used multi-objective PSO method for optimization of selected GEP models. Our GEP-PSO optimized models could give us intact optimized formula for proliferation of Chandler and Rayen (Table 3).

The mono-objective GA optimized MLPNN and RBFNN-based models obtained in our previous studies [20, 24] on Pyrodwarf and OHF *Pyrus* rootstocks showed the significance of some minerals such as NH_4_^+^ and NO_3_^−^ and/or PGRs for explants proliferation. Our previous research [25] on G × N15 *Prunus* rootstock by using mono-objective GA optimized ANN models found the higher importance of NO_3_^−^, NH_4_^+^, Ca^2+^, K^+^, and PO_4_^2−^ towards Mg^2+^, Cl^−^ and SO_4_^2−^ for in vitro proliferation. Our recent study [28] on *Pyrus* rootstocks using mono-objective GA optimization of GEP models indicated that high PR may cause low quality plantlets. In accordance with it, our study [25] on G × N15 using mono-objective GA optimization of ANN models also predicted that increasing the NH_4_^+^ concentration will enhance shoot number and length with higher number of non-healthy shoots but decreasing amount of NH_4_^+^ will enhance the plantlets quality. Our results [28] on pear rootstock using RBFNN and GEP modeling procedures also indicated that a lower content of nitrogen will result in higher quality plantlets. NH_4_^+^, NO_3_^−^ and K^+^ interaction has been the main subject of most in vitro studies [62] but using ML models, [63] reported interaction of K^+^, EDTA^−^ and SO_4_^2−^ with critical effect of K^+^ on PR of pistachio; as low and high concentrations of K^+^ resulted in the highest and lowest PR, respectively. Study on *Prunus* sp. also showed that K^+^ at low concentration promotes PR [64]. Nezami-Alanagh et al. [63] concluded that either low or too high amounts of K^+^, EDTA^−^ and SO_4_^2−^ ions result in low quality plantlets. Considering macro- and micro-elements, our multi-objective PSO optimized GEP models in Chandler showed that increasing NH_4_^+^, NO_3_^−^ and SO_4_^2−^ increased PR and Vit while decreasing CW and STN. But the results in Rayen showed that increasing SO_4_^2−^ except K_2_SO_4_ as well as increasing NO_3_^−^ except KNO_3_ increased PR and CW while decreasing STN and Vit (Table 3).

Reed et al. [65] emphasized on the optimization of nitrogen components content of the culture media to stimulate high number of elongated shoots and reduced amount of callus, in different pear species. Nezami-Alanagh et al. [66] suggested avoiding high content of NH^+^ to reduce callus formation in the in vitro pistachio shoots. Low amounts of some of the MS medium components such as KNO_3_, MgSO_4_, KH_2_PO_4_, CaCl_2_, and NH_4_NO_3_ have been reported to contribute to STN promotion in some *Pyurus* species [67]. Whereas based on our results, lower concentrations of K_2_SO_4_, MgSO_4_, MnSO_4_, CuSO_4_ in Chandler and K_2_SO_4_ and KNO_3_ in Rayen reduced the occurrence of STN. The results of [63] using neurofuzzy logic showed that low amount of K^+^ and mid to high concentrations of SO_4_^2−^ inhibit the STN in pistachio explants with lower signs in UCB1than in Ghazvini which refers to the genotypes differences as we found in our study. Ion confounding problem again prevents determining exact relationship between a given mineral and the physiological disorder.

The neurofuzzy logic procedure show a linear positive effect of nicotinic-acid and pyridoxine–HCl on pistachio parameters of shoot multiplication [68], but, to our knowledge, there is no study about the impact of vitamins on the proliferation of walnut. Nezami-Alanagh et al. [66] showed that the glycine and thiamin-HCl affected differently on some in vitro disorders of pistachio. They showed that increasing glycine content highly reduced the development of callus. Our study showed that higher content of vitamins reduced CW in Chandler (Table 3) and reduced vitamins content in Rayen which caused higher CW (Table 3). Rayen was more recalcitrant to micropropagation than Chandler, hence, achieving higher PR and lower incidence of STN and Vit can cover the low increase in CW.

Genotype is an important factor influencing the occurrence of physiological disorders in walnut which is in agreement with reports of [63, 66] in pistachio. Similarly, other researches on pear [20, 24, 28, 67] explained that the in vitro physiological disorders incidence caused by unbalanced mineral nutrition differed among genotypes.

The purpose of our study was to present an ML approach with high accuracy for prediction of optimized culture media. We applied techniques of MLPNN, KNN and GEP combined with PSO to walnut proliferation data sets to achieve the most appropriate proliferation results. Comparison of our results with the previous ones [20, 24–28, 63, 64, 66] indicates that using at least two methods together results in more precise consequences. So that, comparing the results of the used methods showed the effect of media components enhancing or reducing the measured parameters (Table 3). The efficiency of the developed optimized media was compared to DKW. The media constituents proposed by our PSO optimized GEP models related to Chandler showed that decrease in K_2_SO_4_, MgSO_4_, MnSO_4_, CuSO_4_ and BAP besides increase in other nutrients, PGRs and vitamins increased PR as well as Vit while reducing CW, and STN. Nevertheless, it was slightly different for Rayen as decrease in K_2_SO_4_, KNO_3_, vitamins and BAP along with increase in remained nutrients and PGRs caused higher PR and CW but lower STN and Vit (Table 3). The use of macro- and micro-nutrients as factors, in many micropropagation studies [20, 24, 28, 63, 68], indicates the ion confounding problem, being problematic to recognize precisely corresponding ion(s) affecting the studied parameter [69]. Our results in comparison to previous studies on walnut [11, 15, 37–39] which were about minerals and/or PGRs effects, showed for the first time that not only the effects of minerals depend on the used PGRs concentration but vitamins concentration affects the explant response. The interaction of minerals, PGRs and vitamins could determine the quantity and quality of proliferated plantlets. The plant species and genotype are also highly important in predicting the explant growth response to the minerals, PGRs and vitamins interaction.

Plant PGRs interactions make a critical complication in regulating the processes of plant growth, as well. Cytokinin controls cell proliferation [70] and auxin enhances the sensitivity of apical meristem less mitotically active cells to cytokinin [71]. Cytokinin to auxin ratio is a key signal which controls phenotype [72]. As auxin and cytokinin have roles in DNA replication and cell cycle regulation, respectively [73]. PGRs effects may vary with plant species. Ref. [26] results on *Prunus* rootstock indicated that applying cytokinin and auxin together will result in higher PR than employing each one alone. According to their results, PGRs concentration and interaction are also important. According to these results and [74] and [75] findings, we used various concentrations of BAP, TDZ and IBA in our experiments. Our adverse results can be attributed to the interaction of genotype and culture medium constituents [76] with PGRs [20]. Type and concentration of cytokinin highly affected in vitro growth and survival of black walnut [39]. Ref. [37] reported that lower concentrations of zeatin was better than BAP for fast shoot elongation of black walnut nodal explants, while higher levels of zeatin and BAP led to shoot necrosis. Using TDZ at 0.01–0.02 mg/l in the medium resulted in an enhanced rate of morphological disorders [37]. But higher levels of TDZ (1.30 and 0.52 mg/l in Chandler and Rayen, respectively) in our present study resulted in reduction of STN in both Chandler and Rayen. *Juglans regia* was successfully micropropagated using 0.1–2.01 mg/l BAP [4, 8, 12, 77–81]. Our used BAP concentrations (0.67 and 0.99 mg/l for Chandler and Rayen, respectively) are also in this range. There is no result in the literature about the effect of BAP on the incidence of walnut in vitro physiological disorders. But according to the results of in vitro studies on other plant species like pistachio [64, 82, 83], addition of adequate amount of BAP strongly decreases the incidence of STN.

Therefore, in the present study, we evaluated the interaction of cytokinin and auxin PGRs and medium components including nutrients and vitamins on proliferation of walnut to achieve the most efficient protocol with a reasonable range of PGRs. Our analyses using PSO optimized GEP modeling technique showed that this method can be used as an efficient procedure for evaluating the interaction of different factors on walnut explant growth indices in proliferation phase. Therefore, for the first time GEP is introduced as a great tool in optimizing higher quality and efficiency walnut tissue culture protocols in less time.

Callus development during explant proliferation is a common problem in walnut micropropagation which has been reduced here by increasing PR in Chandler while enhanced by increasing PR in Rayen (Table 3). Yegizbayeva et al. [15] reported that callus formation is not correlated with PR in walnut. Callus formation has been attributed to certain concentrations of different mineral nutrients in various plant species like KH_2_PO_4_, CaCl_2_ and MgSO_4_ in some *Prunus* cultivars [67], NO_3_^−^ in germplasms of *Robus* [84] or MgSO_4_ in *Prunus armeniaca* [85]. Akin et al. [86] reported NH_4_^+^ and after that genotype and SO_4_^2−^ as significant factors affecting callus formation in hazelnut in vitro proliferation using CHAID analysis. Nezami-Alanagh et al. [63] using neurofuzzy logic predicted that high and low concentrations of Fe^2+^ and SO_4_^2−^, respectively, result in the lowest callus formation in pistachio rootstocks explants. They suggested that lower concentration of SO_4_^2−^ in MS reduces shoot tip necrosis and callus development in pistachio in vitro proliferation. While our results showed that lower concentrations of both FeEDDHA and minerals containing SO_4_^2−^ in DKW caused lower CW in both Chandler and Rayen. Bosela et al. [37] showed that the high-salt media i.e. DKW and MS resulted in lower Vit vs. WPM and 1/2X DKW media in walnut.

## Conclusions

Walnut micropropagation is a problematic process with lots of in vitro drawbacks including necrosis, callusing and vitrification. The present study demonstrated the efficiency of plant in vitro proliferation predictive models by using advanced ML modeling procedures. Therefore, a regression model i.e. MLR and three advanced ML models including MLPNN, KNN, and GEP were constructed to predict walnut in vitro PR and associated physiological disorders under the effect of culture medium constituents and genotype. According to the results, following conclusions and suggestions are presented:

- Advanced computational models are the highly precise approaches which can be applied to control and predict walnut explant in vitro performance. They can also be employed as an alternative technique for linear regression and usual statistical analysis methods with noteworthy performance among them. The KNN model has been used for the first time in this study for predicting plant in vitro performance. The optimized models should be applied to predict walnut PR in experimental designs for controlling undesirable physiological disorders.
- All ML models performed accurately for forecasting PR, CW, STN and Vit. Nevertheless, the accuracy of the GEP models were mostly higher than ANN and KNN models. So, the GEP models were selected to be optimized by PSO technique in order to achieve optimal culture media.
- Using above-mentioned ML models is extremely useful for reducing time and cost for formulating efficient walnut tissue culture media.
- The ML-designed media for walnut can not only raise PR (especially about Chandler) but, simultaneously, reduce CW, STN and Vit.
- Genotype is a very important factor which affects the in vitro performance and based on our results, it seems that Rayen as a not bred genotype is more recalcitrant to in vitro propagation than the bred cultivar Chandler.
- Other factors such as sucrose along with our studied medium components and their interaction on PR and occurrence of physiological disorders also need to be incorporated into the predicting model to control the PR comprehensively.

## Methods

MLR, MLPNN, KNN and GEP modeling techniques were applied to make models using various arrangements of minerals, vitamins and PGRs with different concentrations as inputs and different proliferation indices as outputs. The selected models were used to achieve the optimized models using PSO. Two case studies were done using walnut cultivar Chandler and genotype Rayen which have explained details of the used procedures to understand the optimized inputs combinations as follows.

### Case studies

*In vitro* established nodal cultures of Chandler and Rayen were sub-cultured in altered DKW media supplemented with various auxin and cytokinin PGRs concentrations, 30 g/l sucrose and 3 g/l Gelrite. The media were dispersed into jam jars (250 ml) with polyethylene caps after adjusting pH to 5.5. Then, the distributed media were autoclaved for 15 min at 1 kg cm^−2^ s^−1^ (121 °C). The cultures were kept under 16-h white fluorescent (80 µmol m^2^ s^−1^) light at 25 ± 2 °C for 30 days. Subsequently, parameters comprising PR, CW, STN and Vit were measured. In each experiment set, every treatment included 8 replicates (jam jars) for both Chandler and Rayen.

### Taguchi experimental design for optimization of explant proliferation

Taguchi design is a strong and effective tool for the process of optimization that functions constantly and optimally through different conditions. Evaluating numerous factors with limited runs is possible via Taguchi designs i.e. orthogonal arrays. In this design, factors are not weighted more or less in the same experiment and therefore all factors are analyzed independently to each other. Deviation of a product efficient characteristics from their target values is produced by some noise factors such as human errors. Based on orthogonal arrays of Taguchi’s, a standard orthogonal array L_27_ (3^5^) 27 experiments by 26^◦^ of freedom were applied for each of Chandler and Rayen to evaluate the effect of nine factors according to Table 4, on PR, CW, STN, and Vit. For each experiment, three different levels of factor variations were based on various coefficients × DKW basal medium nutrients and different PGRs concentrations (Tables 5). Every nutrient and PGR concentration treatment includes at least 8 replicates. 157 experimental sets (70% of data lines) among 224 sets were randomly chosen for training the modeling methods and the rest 67 sets (30% of data lines) were applied for testing the model’s generalization capacity. In all ML models, k-fold (k = 10) cross validation method [87, 88] was used for training to maintain and grantee the generalizability of constructed models.

**Table 4:** The components and levels of factors used for walnut micropropagation and measured traits mean values applied to characterize it

Walnut	Culture medium	Nutrients (× DKW basal medium) and PGRs concentrations									PR	CW (g)	STN	Vit
		F1	F2	F3	F4	F5	F6	F7	F8	F9				
		NH4NO_3_, CaNO_3_, ZnNO_3_	KNO_3_	K2SO_4_	MgSO_4_, MnSO_4_, CuSO_4_	KH2PO_4_, H3BO_3_, Na_2_MoO_4_	FeEDDHA	Thiamine, Nicotinic acid, Glycine	BAP (mg/l)	TDZ, IBA (mg/l)				
Chandler														
	1	0.5	2	2	2.5	0.5	0.5	0.5	2	2	9.5 ± 0.18	0.79 ± 0.01	30.5 ± 0.9	17.3 ± 0.32
	2	1.25	2	0	1.5	1.5	2	0.5	0.5	1.25	8.1 ± 0.44	0.20 ± 0.01	20.3 ± 0.6	11.7 ± 0.36
	3	2	2	1	0.5	2.5	1.25	0.5	1.25	0.5	6.75 ± 0.2	0.49 ± 0.02	27.2 ± 0.4	13.1 ± 0.44
	4	2	1	0	2.5	1.5	0.5	2	2	1.25	10 ± 0.32	0.89 ± 0.05	31.2 ± 1.1	12.8 ± 0.29
	5	1.25	0	1	2.5	1.5	2	0.5	1.25	2	8.6 ± 0.18	0.97 ± 0.06	33.3 ± 1	21.2 ± 0.45
	6	2	0	2	1.5	2.5	1.25	0.5	2	1.25	7.3 ± 0.7	1.09 ± 0.06	30.1 ± 0.4	29.1 ± 0.51
	7	1.25	2	0	1.5	2.5	0.5	1.25	1.25	2	6.2 ± 0.31	0.89 ± 0.01	30.7 ± 0.7	16.5 ± 0.32
	8	0.5	2	2	2.5	1.5	1.25	1.25	0.5	0.5	10.5 ± 1.1	0.28 ± 0.01	20.2 ± 0.9	0
	9	0.5	1	1	1.5	0.5	0.5	0.5	1.25	1.25	6.3 ± 0.3	0.5 ± 0.01	21 ± 0.8	0
	10	2	2	1	0.5	1.5	0.5	2	0.5	2	10.7 ± 0.9	0.99 ± 0.03	36.1 ± 1.1	11.5 ± 0.42
	11	0.5	0	0	0.5	1.5	1.25	1.25	1.25	1.25	11.7 ± 1	0.5 ± 0.01	33.1 ± 0.9	5.5 ± 0.32
	12	1.25	1	2	0.5	1.5	2	0.5	2	0.5	10.5 ± 0.3	0.49 ± 0.01	39.3 ± 1.1	10.7 ± 0.36
	13	1.25	1	2	0.5	1.5	2	0.5	2	0.5	10 ± 0.32	0.49 ± 0.01	34.3 ± 0.8	6.7 ± 0.35
	14	0.5	0	0	0.5	0.5	0.5	0.5	0.5	0.5	4.8 ± 0.3	0.24 ± 0.01	20.6 ± 0.7	4.6 ± 0.37
	15	1.25	0	1	2.5	0.5	1.25	2	0.5	1.25	7.6 ± 0.18	0.2 ± 0.01	12.6 ± 0.6	9.8 ± 0.39
	16	0.5	0	0	0.5	2.5	2	2	2	2	5.7 ± 0.31	0.89 ± 0.01	31.6 ± 0.8	27.1 ± 0.45
	17	2	1	0	2.5	0.5	2	1.25	1.25	0.5	9.7 ± 0.28	0.13 ± 0.01	13.3 ± 0.3	13.2 ± 0.33
	18	0.5	1	1	1.5	2.5	2	2	0.5	0.5	6.7 ± 0.25	0.18 ± 0.01	11.3 ± 0.3	3.8 ± 0.29
	19	1.25	1	2	0.5	0.5	1.25	2	1.25	2	8 ± 0.32	0.52 ± 0.01	27.1 ± 0.5	6.8 ± 0.25
	20	1.25	1	2	0.5	2.5	0.5	1.25	0.5	1.25	7.3 ± 0.41	0.20 ± 0.01	15 ± 0.6	6.8 ± 0.39
	21	0.5	1	1	1.5	1.5	1.25	1.25	2	2	5.8 ± 0.3	0.73 ± 0.01	22 ± 0.5	9.3 ± 0.32
	22	1.25	0	1	2.5	2.5	0.5	1.25	2	0.5	9.1 ± 0.29	0.2 ± 0.01	15.7 ± 1	7.1 ± 0.29
	23	2	2	1	0.5	0.5	2	1.25	2	1.25	5.8 ± 0.3	1.1 ± 0.03	21.8 ± 0.6	19.5 ± 0.32
	24	0.5	2	2	2.5	2.5	2	2	1.25	1.25	21.8 ± 0.7	0.09 ± 0.01	11.1 ± 0.7	3.1 ± 0.12
	25	2	0	2	1.5	0.5	2	1.25	0.5	2	13.6 ± 0.3	0.98 ± 0.01	18.1 ± 0.8	10.7 ± 0.45
	26	2	1	0	2.5	2.5	1.25	0.5	0.5	2	12.1 ± 0.3	0.8 ± 0.02	13.6 ± 0.6	9.7 ± 0.44
	27	2	0	2	1.5	1.5	0.5	2	1.25	0.5	9.7 ± 0.31	0.51 ± 0.01	11.6 ± 0.6	29 ± 0.46
	DKW	1	1	1	1	1	1	1	1	0.1	7.12 ± 0.35	0.3 ± 0.03	10.75 ± 0.45	0
Rayen														
	1	0.5	2	2	2.5	0.5	0.5	0.5	2	2	8.5 ± 0.42	0.92 ± 0.03	33.8 ± 0.8	21.7 ± 0.59
	2	1.25	2	0	1.5	1.5	2	0.5	0.5	1.25	7.7 ± 0.31	0.28 ± 0.02	25.6 ± 1.1	14 ± 0.26
	3	2	2	1	0.5	2.5	1.25	0.5	1.25	0.5	6.3 ± 0.37	0.48 ± 0.01	49.5 ± 1.4	15.7 ± 0.36
	4	2	1	0	2.5	1.5	0.5	2	2	1.25	8 ± 0.37	0.94 ± 0.02	25.3 ± 1.5	16.8 ± 0.29
	5	1.25	0	1	2.5	1.5	2	0.5	1.25	2	7.7 ± 0.31	0.99 ± 0.02	27.2 ± 1.3	27.7 ± 0.45
	6	2	0	2	1.5	2.5	1.25	0.5	2	1.25	8 ± 0.32	0.79 ± 0.03	29.6 ± 0.6	31.3 ± 0.54
	7	1.25	2	0	1.5	2.5	0.5	1.25	1.25	2	5.8 ± 0.29	1.07 ± 0.04	31.2 ± 1	17.3 ± 0.37
	8	0.5	2	2	2.5	1.5	1.25	1.25	0.5	0.5	6.3 ± 0.26	0.31 ± 0.01	21.8 ± 1.1	6 ± 0.46
	9	0.5	1	1	1.5	0.5	0.5	0.5	1.25	1.25	6.3 ± 0.46	0.52 ± 0.01	27 ± 0.4	6.2 ± 0.36
	10	2	2	1	0.5	1.5	0.5	2	0.5	2	7 ± 0.53	1.05 ± 0.04	33.1 ± 1.2	15 ± 0.37
	11	0.5	0	0	0.5	1.5	1.25	1.25	1.25	1.25	8 ± 0.42	0.45 ± 0.01	28.5 ± 0.4	8 ± 0.26
	12	1.25	1	2	0.5	1.5	2	0.5	2	0.5	8.2 ± 0.49	0.17 ± 0.01	28 ± 0.6	10.6 ± 0.46
	13	1.25	1	2	0.5	1.5	2	0.5	2	0.5	9 ± 0.46	0.39 ± 0.01	27.3 ± 0.9	9.2 ± 0.36
	14	0.5	0	0	0.5	0.5	0.5	0.5	0.5	0.5	6.1 ± 0.39	0.27 ± 0.01	23.3 ± 0.5	4 ± 0.26
	15	1.25	0	1	2.5	0.5	1.25	2	0.5	1.25	7 ± 0.26	0.29 ± 0.01	21.7 ± 1.3	12.1 ± 0.29
	16	0.5	0	0	0.5	2.5	2	2	2	2	6 ± 0.46	0.72 ± 0.02	29 ± 1.5	31.8 ± 0.44
	17	2	1	0	2.5	0.5	2	1.25	1.25	0.5	7.3 ± 0.37	0.26 ± 0.01	18.1 ± 1.4	16.1 ± 0.37
	18	0.5	1	1	1.5	2.5	2	2	0.5	0.5	6.3 ± 0.53	0.31 ± 0.01	27.2 ± 1.3	5 ± 0.32
	19	1.25	1	2	0.5	0.5	1.25	2	1.25	2	7.5 ± 0.5	0.46 ± 0.01	27.8 ± 0.5	7.1 ± 0.29
	20	1.25	1	2	0.5	2.5	0.5	1.25	0.5	1.25	7.5 ± 0.32	0.29 ± 0.01	20.1 ± 0.6	6.7 ± 0.25
	21	0.5	1	1	1.5	1.5	1.25	1.25	2	2	6 ± 0.26	0.94 ± 0.02	27.1 ± 0.7	10.7 ± 0.36
	22	1.25	0	1	2.5	2.5	0.5	1.25	2	0.5	6.5 ± 0.32	0.16 ± 0.01	22 ± 0.9	6 ± 0.37
	23	2	2	1	0.5	0.5	2	1.25	2	1.25	6.6 ± 0.25	0.91 ± 0.03	27.2 ± 0.9	22.5 ± 0.5
	24	0.5	2	2	2.5	2.5	2	2	1.25	1.25	7 ± 0.31	0.17 ± 0.01	27.5 ± 0.6	6 ± 0.26
	25	2	0	2	1.5	0.5	2	1.25	0.5	2	10.2 ± 0.28	0.55 ± 0.02	21.6 ± 0.8	9.3 ± 0.41
	26	2	1	0	2.5	2.5	1.25	0.5	0.5	2	7.2 ± 0.36	0.94 ± 0.03	29.5 ± 0.7	11.6 ± 0.37
	27	2	0	2	1.5	1.5	0.5	2	1.25	0.5	8.3 ± 0.37	0.46 ± 0.01	27.3 ± 0.3	35 ± 0.46
	DKW	1	1	1	1	1	1	1	1	0.1	6.25 ± 0.4	0.5 ± 0.06	23.75 ± 0.59	16.12 ± 1.1

**Table 5:** The components of factors and experimental runs ranges based on DKW medium

Code of Factors	Culture medium components	Level of factors
X1	NH_4_NO_3_, CaNO_3_, ZnNO_3_	0.5–1.25–2×
X2	KNO_3_	0–1–2×
X3	K_2_SO_4_	0–1–2×
X4	MgSO_4_, MnSO_4_, CuSO_4_	0.5–1.5–2.5×
X5	KH_2_PO_4_, H_3_BO_3_, Na_2_MoO_4_	0.5–1.5–2.5×
X6	FeEDDHA	0.5–1.25–2×
X7	Thiamine, Nicotinic acid, Glycine	0.5–1.25–2×
X8	BAP	0.5–1.25–2 mg l^−1^
X9	TDZ, IBA	0.5–1.25–2 mg l^−1^

## Modeling techniques

### Multiple linear regression

MLR analysis is a multivariate statistical method to assess the relationship between multiple independent variables and an individual dependent variable. Two important purposes of MLR are prediction and explanation. The MLR prediction comprises the level to which the independent variables can predict the dependent variables. The mentioned description of MLR estimates the coefficients of regression, their sign, magnitude and statistical interface, for each independent variable [89]. Linear regression is considered as the first statistical method in regression and assumed to be an index technique to be used by new methods. As other regression methods, the relationships between a dependent variable and multiple independent variables are modeled by MLR and a linear equation is fitted to the experimental data. MLR technique makes relationship between independent variable k value and the dependent variable M value. The regression equation of n input variables × 1, × 2, …, kn is according to the following:

$$M=\alpha 0+ \alpha 1k1+\dots + \alpha n$$

in which the dependent variable is M, k (× 1, …, kn) denotes a vector of input variables, α0 indicates intercept (a constant), and α is the coefficient of regression vector, each of which is for each expository variable. Y experimental values have various meanings and are supposed with the identical standard deviation ε. The SPSS 19 software package was used for the MLR modeling.

### Multilayer perceptron neural network

The neural network is divided into various types based on the transfer functions basis. In the present study, we used multi-layer perceptron (MLPNN) network. The MLPNN model is the most common and widely used type of artificial neural network [90]. This model generally contains an input layer and an output layer. One or more hidden layers can be placed between these two layers. Each neuron in this structure has a number of inputs and a number of outputs. A neuron calculates its output responses based on the weighted sum of all its inputs, performed by a stimulus or transmission function. In the MLPNN model, starting from the input information in the first layer (independent variables), the information flows in only one direction and enters the output layer (dependent variable) by transferring from the hidden layer. The training process of MLPNN model involves adjusting and modifying the weights of the interface between neurons using different network training methods [91]. In this study, Broyden-Fletcher-Goldfarb-Shanno (BFGS) training algorithm has been used. Also, stimulus functions; The tangent hyperbolic (Tanh), sigmoid function (Logs), exponential function (Exp), relu function (Relu) in the hidden layer and linear function (Idn) in the output layer were compared and evaluated and the best function was selected. The number of hidden layers was also determined by trial and error by reaching the minimum error rate. See [91, 92] for more information.

### *k*-nearest neighbors’ algorithm

The *k*-nearest neighbors (KNN) model is a non-supervised learning machine algorithm for data classification. In this model, each data represents a coordinate position in a vector-space model that the information of each particular section must have similar properties as well as be close to each other. In the KNN algorithm, determining the number of neighbors (k) as well as the method based on which the distance between them is calculated is of particular importance. If k is considered too small, then neighboring points that do not appear in the classification will reduce the accuracy of the results. On the other hand, if k is considered too large, the results of the same classifications may be merged as the computational volume increases [93]. The nearest neighbor was evaluated and selected from different values ​​to find the best value of k and to achieve the highest model accuracy. Distances between neighboring points were determined using various geometric methods. In this study, the methods of Euclidean Distance, Chebyshev Distance, Manhattan Distance and Minkowski Distance were studied and the best method was selected.

### Gene expression programming

GP is a modeling approach used to model the structural engineering complications behavior. It is an extension of genetic algorithm that utilizes a program space for searching, rather than using a data space. An important benefit of applying GP-based techniques toward other methods is their capability to produce equations of prediction without using any hypothesis for previous relationship form. Many researchers have applied GP and GP-based methods to find any complicated relationships fitting different experimental data [44, 94, 95]. GEP has been introduced as an effective substitute method to the conventional GP [31, 46]. GEP have established many computer programs, by getting encoded in linear chromosomes with constant length, each of which included several encoding genes [31, 96]. GEP is originated of evolutionary algorithms such as GA and GP. In this technique, an individual population is applied and afterwards, fitness function and genetic variations are used to select better individuals. The genetic variations are presented by genetic operators. GEP is a learning machine which is assumed to learn the variables relationship in datasets. The individual programming technique is different in GEP and its predecessors GP and GA since GEP programs individuals as linear strings (chromosomes) with fixed length which are finally displayed by expression trees as unsophisticated diagram. While, GP and GA express individuals in the form of linear strings (chromosomes) with fixed length and nonlinear entities of diverse forms (parse trees) and dimensions, respectively. One of the strongpoints of GEP towards GP and GA is that genetic operators run very easily at the level of chromosome in GEP producing genetic diversity creation. Another strength of GEP is its exclusive, multi-genic nature letting more complicated programs with numerous subprograms to be developed. Both GP and GA advantages are collected in GEP, whereas some of their constraints are met [57, 97, 98].

**Fig. 10:**
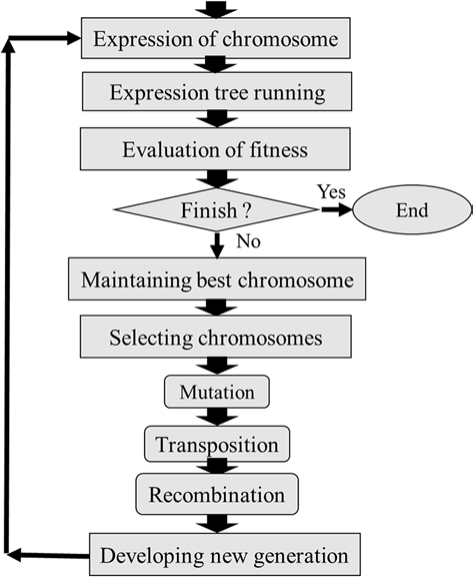
Diagram of gene expression programming as a prediction model

The real GEP chromosome phenotype is the illustration in Fig. 10 and the genotype would be simply described of the phenotype as represented in Eq. (1)

1 $$\left(a+b\right)*(c-d)$$

Functional steps of the GEP are represented in Fig. 10 [31]. According to this diagram, the GEP start point is a population of chromosomes. After that, the chromosomes genes are expressed, and each individual fitness is analyzed. Then, the individuals are defined according to their fitness to reproduce with alteration. The same development process is run on the new individuals’ generation. Overall, this technique is replicated for a particular number of generations or it is performed until reaching a termination condition. Roulette wheel sampling with elitism is employed by GEP system to ensure that the top individuals, according to the fitness, are remained and copied to the next generation. Once genetic operator(s) are performed on chosen chromosomes, comprising mutation, cross over and rotation, diversity is developed into the population.

The GeneXpro software package was applied to perform the GEP models. The parameters employed in the GEP models are represented in Table 6.

**Table 6:** Parameters of training GEP model

Parameter	Parameter description	Parameter setting
1	Function set	+ , −, ÷ , *, sin, cos, x^2, x^3, power,
2	Chromosomes	50
3	Head size	10
4	Number of genes	4
5	Linking function	addition
6	Fitness function	Root relative square error (RRSE)
7	Mutation rate	0.044
8	Inversion rate	0.1
9	One-point recombination rate	0.1
10	Two-point recombination rate	0.3
11	Gene recombination rate	0.1
12	Gene transportation rete	0.1

In this study, the selected functions and mathematical operators are rational and not definite so that the plant modeling designer is free to select such functions according to the studied problem anatomy. The functions and operators were selected with a viewpoint of invocating simpleness of the advanced model assuring quicker convergence. The size of the population (chromosomes number) adjusts the programs number into the population. The larger the population, the longer it takes for an iteration run. High chromosomes number were tried to realize minimum error models. The program running continued to reach no significant rectification in the models’ performance. Here, we aimed to achieve obvious relationship between decision variables and response variables. GEP clear formulations were obtained for PR, CW, STN and Vit as a function of experimental parameters including Y1, Y2, Y3 and Y4 = f (X1, X2, X3, X4, X5, X6, X7, X8 and X9) (Table 1).

Input data were normalized in the range of 0 and 1 according to the Eq. 2:

2 $${X}_{n}=\frac{{X}_{i}-{X}_{min}}{{X}_{max}-{X}_{min}}$$

where *X*_*n*_ is normalized dimensionless data, *X*_*i*_ is observed data, *X*_*min*_ is the minimum amount of observed data, and *X*_*max*_ is its maximum value.

### Comparison of the performance of developed models

To evaluate the precision of created models, we used different statistical indices including coefficient of determination (R^2^), root mean square error (RMSE) and mean absolute error (MAE) based on Eqs. 5, 6 and 7:

5 $${R}^{2}=\frac{{\left({\sum }_{i=1}^{N}\left({y}_{i}-\overline{\widehat{y} }\right)\left(\widehat{{y}_{i}}-\overline{\widehat{y} }\right)\right)}^{2}}{{\sum }_{i=1}^{N}{\left({y}_{i}-\overline{y }\right)}^{2}{\sum }_{i=1}^{N}{\left(\widehat{{y}_{i}}-\overline{\widehat{y} }\right)}^{2}}$$

6 $$RMSE=\sqrt{\frac{1}{N}{\sum }_{i=1}^{N}{\left({y}_{i}-\widehat{y}\right)}^{2}}$$

7 $$MAE=\frac{1}{N}{\sum }_{i=1}^{N}\left|{y}_{i}-\widehat{y}\right|$$

where $y$ and $\overline{y }$ are observed values and their mean and $\widehat{y}$ and $\overline{\widehat{y} }$ are predicted values and their mean, respectively, as well for N samples. Analyses of parameters were performed together to achieve an accurate medium composition.

In addition, the predicted values by the developed models were plotted against the corresponding observed values to evaluate the ability of models for prediction.

### Particle swarm optimization of GEP models

PSO is a method of evolutionary calculation and swarm intelligence algorithm according to population to solve the pervasive problem of optimization that was developed by [99]. It is a method of mathematical computation that starts with the swarm (a population of grain) and mostly based on social models, such as the swarm theory, fish schooling and bird flocking [20]. PSO key factors are with behavior of swarm i.e. keeping optimum distances between different members and their neighbors. To optimize each particle location, their position is modified as arranged for the objective function within the search area. Thus, PSO key factor is a particle velocity which is compared to the previous one in each repetition to lead the particle to its optimal position. The best solution (fitness) every particle in a swarm achieves so far in each repetition, named pbest. Extra “best” value that a particle is attained in the population up to now followed by the particle swarm optimizer which is global best, named gbest. Each particle velocity in a swarm is estimated by Eqs. 3 and 4 [99].

3 $${V}_{i+1}=w{V}_{i}+{c}_{1}{r}_{1}\left({pBest}_{i}-{X}_{i}\right)+{c}_{2}{r}_{2}({gBest}_{i}-{X}_{i})$$

4 $${X}_{i+1}={X}_{i}+{V}_{i+1}$$

in which, *V*_*i*+*1*_ is each particle new velocity based on prior velocity (*V*_*i*_), *w* is inertial coefficient (0.8–1.2), *c*_*1*_ and *c*_*2*_ are cognitive and social coefficients, respectively (0–2), *r*_*1*_ and *r*_*2*_ are random values for each velocity update (0–1) and *X*_*i*+*1*_ is new location for each particle according to the prior location (*X*_*i*_).

## Data Availability

The datasets used and/or analyzed during the current study are available from the corresponding author on reasonable request.

## Abbreviations

PGRs: Plant growth regulators
STN: Shoot tip necrosis
Vit: Vitrification
MLL: Machine learning
MLPNN: Multi-layer perceptron neural network
KNN: *k*-Nearest neighbors
GEP: Gene expression programming
MLR: Multiple linear regression
PSO: Particle swarm optimization
DKW: Driver and Kuniyuki walnut
ANN: Artificial neural network
GA: Genetic algorithm
RBFNN: Radial basis function neural network
GP: Genetic programming
MOEA: Multi-objective evolutionary optimization algorithm
MOPs: Multi-objective optimization problems
PR: Proliferation rate
CW: Callus weight
MS: Murashige and Skoog
